# The efficacy of CRISPR-mediated cytosine base editing with the RPS5a promoter in *Arabidopsis thaliana*

**DOI:** 10.1038/s41598-021-87669-y

**Published:** 2021-04-13

**Authors:** Minkyung Choi, Jae-Young Yun, Jun-Hyuk Kim, Jin-Soo Kim, Sang-Tae Kim

**Affiliations:** 1 Center for Genome Engineering, Institute for Basic Science, Daejeon, 34126 Republic of Korea; 2 Institute of Green Bioscience & Technology, Seoul National University, Pyeongchang, Republic of Korea; 3 Department of Medical and Biological Sciences, The Catholic University of Korea, Bucheon, 14662 Republic of Korea

**Keywords:** Plant molecular biology, Genetic engineering

## Abstract

CRISPR/Cas9-mediated genome editing is an important and versatile technology in modern biological research. Recent advancements include base-editing CRISPR tools that enable targeted nucleotide substitutions using a fusion protein comprising a nickase variant of Cas9 and a base deaminase. Improvements in base editing efficiencies and inheritable of edited loci need to be made to make CRISPR a viable system in plants. Here, we report efficiency of cytosine base editors (CBEs) in *Arabidopsis thaliana* by applying the strong endogenous RPS5a promoter to drive the expression of nickase Cas9 and either rAPOBEC1 from rat (BE3) or the PmCDA1 activation-induced cytidine deaminase from sea lamprey (AIDv2). Compared with the strong heterologous CaMV35S promoter of viral origin, the RPS5a promoter improved CBE efficiency by 32% points with the number of T_1_ plants showing over 50% conversion ratio when the *LFY* gene was targeted. CBE induced nonsense mutations in *LFY* via C-to-T conversion, which resulted in loss-of-function *lfy* phenotypes; defects in *LFY* function were associated with the targeted base substitutions. Our data suggest that optimal promoter choice for CBE expression may affect base-editing efficiencies in plants. The results provide a strategy to optimize low-efficiency base editors and demonstrate their applicability for functional assays and trait development in crop research.

## Introduction

Clustered regularly interspaced palindromic repeat (CRISPR) and CRISPR-associated protein 9 (Cas9)-mediated genome editing technologies provide a novel platform for biological research by enabling precise and programmable modification of genetic elements^1–3^. CRISPR/Cas9-mediated genome engineering functions by introducing a DNA double-strand break 3 base pairs (bp) upstream of the protospacer adjacent motif (PAM), to which Cas9 endonuclease is recruited via a single stranded guide RNA (gRNA). The double-strand break is often repaired by the error-prone non-homologous end joining (NHEJ) process; NHEJ can generate a small insertion or deletion (indel) during repair. CRISPR/Cas9-mediated indels often introduce an early stop codon by a frameshift, rendering the target gene non-functional. Although CRISPR/Cas9 is a powerful tool for knocking out genes, typically by generating loss-of-function alleles, introducing specific amino acid changes to a target gene can be difficult due to technical limitations; this has limited functional genomics research in which specific mutations are desired to address sub- or neo-functionalization of a given gene product.

Base editors that can function in eukaryotic cellular systems with various base deaminases fused to CRISPR/Cas9 have recently been developed, thus broadening the gene modification toolbox for functional genomics^4–6^. Cytosine base editors (conversion of C/G to T/A; CBEs, with partial conversion of C/G to G/C unexpected) can introduce base substitutions that lead to amino acid changes or splicing modifications. Two CBE systems, base editor 3 (BE3) and activation-induced cytidine deaminase version 2 (AIDv2) contain the adapted cytidine deaminases rAPOBEC1 from rat and PmCDA1 from sea lamprey, respectively^5,7^⁠. Generally, the base editing target window of BE3 is narrower than that of AIDv2; BE3 has 4–8 positions on the 20 bp target sequences, while AIDv2 has 2–10 positions when counting the canonical PAM sequence (NGG) as at positions 21–23.

CBEs have been applied in various plant species, ranging from monocots such as *Oryza sativa*, to the model plant eudicot *Arabidopsis thaliana* (hereafter, *Arabidopsis).* However, the overall success rate for base editing using CBEs remains relatively low compared to CRISPR/Cas9^8–14^. Parameters affecting genome editing efficiencies in plant cells have been determined using stable transgenic *Arabidopsis* lines, into which the conventional CRISPR/Cas9 cassette was introduced via *Agrobacterium*-mediated transformation^15–17^. Promoter choice is an important factor that affects the indel-inducing efficiencies of Cas9 in plant cells. Both CaMV35S (hereafter 35S) and ubiquitin promoters, which are widely used in plant transformation practices, have resulted in relatively high Cas9 expression in plant cells with moderate success rates. However, recent evidence suggests that alternative promoter systems may be more suitable for CRISPR/Cas9 expression and for generating stable transgenic lines. For example, the ribosomal protein subunit 5a (RPS5a) promoter of *Arabidopsis* has recently been shown to achieve higher efficiencies of adenine base editing and more successful transmission of edited alleles into the next generation in *Arabidopsis* compared to canonical promoters^18^. Similar improvements were also observed in regular CRISPR/Cas9 mediated mutagenesis using the RPS5a promoter^15,16^. However, the effectiveness of the RPS5a promoter for CBEs has not yet been addressed.

In this study, we aimed to test the efficacy of the RPS5a promoter in CBEs and to assess how promoter choice can influence the efficiencies of two CBE systems: BE3 and AIDv2. We selected the *LEAFY* (*LFY*) gene, a master transcription factor for flowering, as a CBE target to address the efficiency of CBE-mediated base editing in *Arabidopsis*. Disruption of *LFY* function can be easily identified by characteristic knock-out phenotypes (*lfy*), which include the loss of floral organs such as petals and stamens^19–21^. We measured the efficiencies of four CBE vector constructs with combinations of two different promoters, RPS5a and 35S, to drive the expression of the two CBE variants; the CBE systems were designed to introduce nonsense point mutations of cytosine (C) to thymine (T) in two target sites within the *LFY* gene. Clear *lfy* mutant phenotypes were observed in both the first (T_1_) and second (T_2_) generations of transgenic lines. A next-generation sequencing (NGS)-assisted large-scale survey of the C-to-T conversion efficiencies in 440 transgenic T_1_ individuals enabled us to assess the base editing patterns of the two target sites, revealing that the RPS5a promoter may affect cytosine base editing efficiencies.

## Results

### The *lfy* mutant phenotype was induced in the primary generation of transgenic *Arabidopsis*

**Figure 1:**
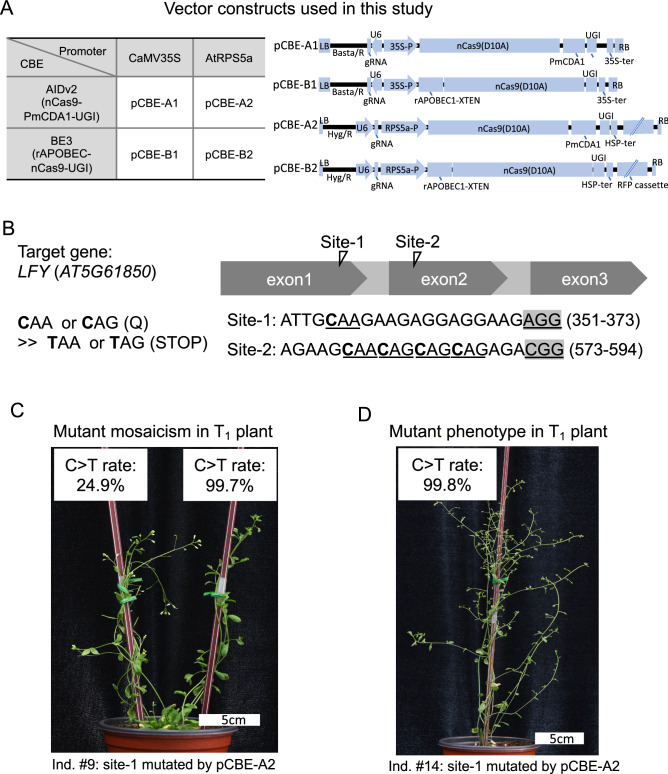
CRISPR/Cas9-mediated cytosine base editing in *Arabidopsis*. (**A**) Summary of constructs used in this study and detailed construct composition for each. *LB* left border, *Basta/R* Basta resistance, *U6* U6 promoter, *UGI* Uracil glycosylase inhibitor, *RB* right border, *HSP-ter* heat shock protein terminator, *Hyg/R* Hygromycin resistance, *RFP* red fluorescent protein. (**B**) All specific cytosine (C) positions targeted in each site are presented in bold and codons are underlined. The nucleotide positions of single guide RNA (gRNA) sites are shown in parentheses. The protospacer adjacent motif (PAM) is underlined. (**C,D**). Examples of T_1_ plants showing *lfy* mutant phenotypes. (**C**) An example T_1_ plant individual transformed with pCBE-A2 targeting site-1, showing the wild-type phenotype (left) and the *lfy* mutant phenotype (right). C-to-T conversion rates were analyzed using tissues collected from the left or right part for targeted deep sequencing. (**D**) A T_1_ individual plant transformed with pCBE-A2 targeting site-1 showing the *lfy* mutant phenotype only. Ind.: individual.

To examine and test the efficacy of base editing tools to introduce the non-sense mutation *in planta*, we selected the plant RPS5a promoter and the viral 35S promoter to generate four different construct combinations with two CBEs: BE3 and AIDv2 (Fig. 1A). Target sites for gRNA were chosen to recapitulate previously described loss-of-function *lfy* mutations; targeting site-1 (5′-ATTG**C**_**5**_AAGAAGAGGAGGAAGAGG-3′) and site-2 (5′-AGAAG**C**_**6**_AA**C**_**9**_AG**C**_**12**_AG**C**_**15**_AGAGACGG-3′) was expected to induce nonsense mutations using a CBE at the amino acid positions Q119* (site-1) and Q189*, Q190*, Q191*, or Q192* (site-2), by converting the corresponding cytosine bases to thymine bases (Fig. 1). Once the hygromycin-selected individual T_1_ plants reached the reproductive phase, we used the mutant *lfy* phenotype as a proxy for CBE efficiency. For the pCBE-A2 construct, in which the RPS5a promoter controlled CBE AIDv2 expression, carrying an gRNA for target site-1 (Fig. 1A), we observed typical *lfy* mutant phenotypes among the T_1_ individuals. Nineteen of 69 selected T_1_ plants (28%) had typical *lfy* phenotypes in some branches (Fig. 1C) or in whole plants (Fig. 1D); the *lfy* phenotypes were characterized by abnormal flowers with no distinct petals and no male floral organs. To quantify genotypic changes to the target cytosine, we employed a targeted deep sequencing method using NGS (also refer to the amplicon deep sequencing). We determined the C-to-T conversion rate by calculating the percentage of amplicon reads that had substituted cytosine for thymine at the target site, and used this as an indicator of base editing efficiency. The C_5_ (the targeting cytosine position 5 when counting the PAM site as positions 21–23) was queried at the site-1 target by amplicon deep sequencing. DNA that was extracted from branches that displayed the regular wild-type flowers of T_1_ plant #9 yielded a C-to-T conversion rate of 24.9%; however, the C-to-T conversion rate was 99.7% for DNA extracted from branches with abnormal flowers or *lfy* mutant phenotypes in the same individual (#9; Fig. 1C). The correlation between the C-to-T conversion rate and the phenotype indicated that partially reduced expression of unmutated *LFY* is sufficient to promote normal floral development, and the loss-of-function phenotype is only observed when mutant copies of the *LFY* gene product are more abundant. In addition, targeted deep sequencing of T_1_ plant #14, which was transformed with pCBE-A2 and presented the *lfy* phenotype across all branches, had a 99.8% C-to-T conversion rate at C_5_ at site-1 (Fig. 1D). Our data confirmed that the intended CBE-induced mutation was introduced, in which an early stop codon was generated at target site-1 by converting glutamine 119 (CAA:Q) into a stop codon (TAA:Stop) and most importantly pCBE-A2 can induce CBE events in the first generation of transformants, conferring a complete loss-of-function phenotype in some T_1_ individuals.

### The inheritance of mutant alleles into the secondary generation of transgenic *Arabidopsis*

**Figure 2:**
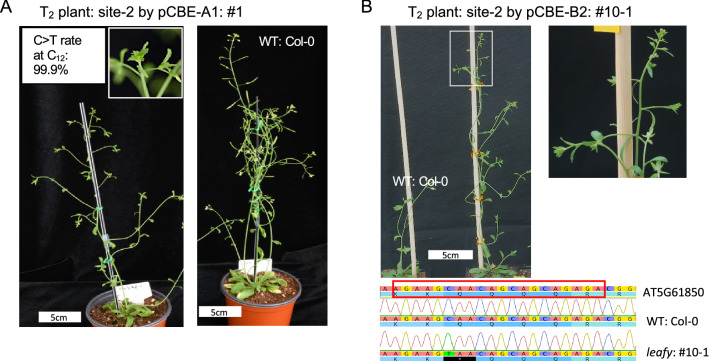
Distinct *lfy* mutant phenotype in T_2_ plants induced by CRISPR/Cas9-mediated cytosine base editing in *Arabidopsis*. (**A**) A T_2_ plant transformed with pCBE-A1 targeting site-2 displaying the *lfy* mutant phenotype, with the wild-type Col-0 plant for comparison. C-to-T conversion was confirmed for position C_12_, which had a conversion rate of 99.9% as calculated following targeted deep sequencing analysis. (**B**) A T_2_ plant transformed with pCBE-B2 targeting site-2 with the Col-0 plant. The meristem region of inlet was magnified next to the whole plant picture. The complete conversion of C-to-T on the position C_6_ was presented by Sanger sequencing result.

To assess the germline transmission of base-edited mutant alleles, we harvested the seeds of T_1_ plants that had been transformed with the pCBE-A1 vector (promoter: 35S, CBE: AIDv2) and the pCBE-B2 vector (promoter: RPS5a, CBE: BE3), which targeted site-2 (5′-AGAAG**C**_**6**_AA**C**_**9**_AG**C**_**12**_AG**C**_**15**_AGAGACGG-3′). Target site-2 has four cytosine residues which, when converted to thymine, introduce early stop codons and thus nonsense mutations in LFY. We measured the C-to-T conversion rates at each possible nonsense mutation position within site-2 using targeted deep sequencing by NGS from individual T_1_ plants that were transformed with pCBE-A1 and pCBE-B2 vectors. The C-to-T conversion rates in six individual T_1_ plants by pCBE-A1 ranged from 18.7 to 63.5% at protospacer position 6 (C_6_), and from 13.2% to 39.6% at position C_12_; no significant changes were observed at the two other possible target cytosines, C_9_ and C_15_, which exhibited C-to-T conversion rates of < 1.9% (Fig. 1B, Table S2). The C-to-T conversion rates in T_1_ plants transformed by pCBE-B2 showed up to 75.5% at position C_6_ but no significant substitutions were found at three other target cytosines (Table S2). We then harvested and sowed seeds from T_1_ individuals showed high conversion rates to screen *lfy* mutant phenotypes in T_2_ plants for both lines transformed by pCBE-A1 and pCBE-B2. In the next generation, 3 of 102 T_2_ individuals by pCBE-A1 presented complete *LFY* knock-out phenotypes, with 99.9% C-to-T conversion rates at position C_12_ only (Fig. 2A); these are from T1 plant with 18.7% C-to-T conversion rate at position C_6_ and 13.3% at C_12_. 2 of 29 T2 individuals from the T1 line with 64.1% C-to-T conversion rate at position C_6_ only by pCBE-B2 also showed complete lfy mutant phenotypes (Fig. 2B). To access germline transmission of mutant alleles induced in the T_1_ generation we checked whether vector plasmids are remained and active for possible ‘de novo’ base editing by carrying out amplification of designated four regions with specific PCR primers on T-DNA vector (Fig. S1). No specific PCR products were detected for three T2 individuals by pCBE-A1 nor for one T2 individual by pCBE-B2. The other individual by pCBE-B2 showed PCR bands for three sets of primers but no band for one set targeting on Cas9 region, which indicates base editing activity is inactive (Fig. S1).

To see possible off-target effect on *lfy* mutant phenotypes in these T_2_ transgenic lines we listed the potential off-target sites allowing up to three mismatches in target sequence using Cas-Offinder^22^ (http://www.rgenome.net). 18 sites were identified; 2 sites containing 2-mismatches, 16 sites containing 3 mismatches with N[A/G]G PAM (5 sites with NGG PAM), and all sites indicated no specific base substitution (Fig. S2).

### Construct comparisons with cytosine to thymine conversion rates in T_1_ plants

**Figure 3:**
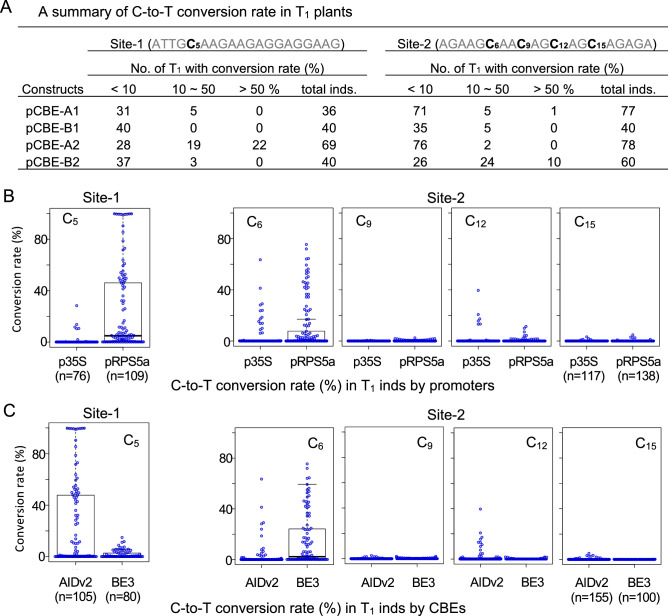
Comparison of the effects of different promoters and base editors on cytosine-to-thymine conversion rates in the first generation of transformed *Arabidopsis* individuals (T_1_). (**A**) C-to-T conversion rates calculated from targeted deep sequencing analysis of each T_1_ individual are presented. The numbers of individuals with low (< 10%), middle (10–50%), or high (> 50%) conversion rates are indicated. (**B**,**C**) Comparison of base editing efficiencies with different combinations of promoter and cytosine base editor (CBE) in vector constructs. All C-to-T conversion rates from T_1_ individual plants were grouped by vector and the construct used and plotted. (**B**) Comparison of conversion rates grouped by promoter set: CaMV35S versus RPS5a (pCBE-A1 and pCBE-B1) versus (pCBE-A2 and pCBE-B2). (**C**) Comparison of conversion rates grouped by CBE set: AIDv2 versus BE3 (pCBE-A1 and pCBE-A2) versus (pCBE-B1 and pCBE-B2). Each open dot represents the C-to-T conversion rate for each T_1_ individual. C-to-T conversion rates were calculated for each targeting cytosine position. *Inds* individuals.

Following validation of *lfy* as a phenotypic marker for functional assays and confirmation of C-to-T conversion activity in our two CBE constructs, we generated more T_1_ lines to perform comparative analyses of promoters and CBE systems. All four CBE vector constructs (Fig. 1A) were introduced into *Arabidopsis* via *Agrobacterium*-mediated transformation by floral dipping, and the C-to-T conversion rates of cytosine targets in individual T_1_ plants were compared using NGS of the two target site amplicons. We found considerable variation in C-to-T conversion rates between each vector construct. For target site-1, which has only one cytosine target at position 5 (C_5_), construct pCBE-A2 (RPS5a-AIDv2) exhibited the highest conversion efficiency of the four constructs tested in the first generation; 32% (22 of 69) of T_1_ individuals carrying pCBE-A2 showed > 50% conversion rates at the target cytosine, while no T_1_ individuals presented > 50% C-to-T conversion rates following transformation with the other CBE constructs (Fig. 3A). For target site-2, we calculated the C-to-T conversion rates of individuals in which all four potential C-to-T base editing targets were converted. One of 60 (1.3%) T_1_ individuals carrying the vector pCBE-A1 (35S-AIDv2) and 10 of 60 T_1_ individuals (16.7%) carrying pCBE-B2 (RPS5a-BE3) showed C-to-T conversion rates > 50% (Fig. 3A).

We then categorized the C-to-T conversion rates by two vector construct factors: the ‘promoter set’ and the ‘CBE set’. For the promoter set, we divided all C-to-T conversion rates into two groups. First, we compared ratios obtained from T_1_ individuals transformed with vectors containing the RPS5a promoter (pCBE-A2 and pCBE-B2) with those from T_1_ individuals carrying the 35S promoter (pCBE-A1 and pCBE-B1). We then grouped C-to-T conversion rates according to the CBE composition; the two groups contained T_1_ individuals transformed with vectors encoding AIDv2 (pCBE-A1 and pCBE-A2) or BE3 (pCBE-B1 and pCBE-B2). T_1_ individuals carrying RPS5a promoter constructs showed higher C-to-T conversion rates at both sites-1 and -2. A total of 13% of T_1_ individuals with the RPS5a promoter (32 of 247) had C-to-T conversion rates > 50%, while only one T_1_ individual with the 35S promoter (n = 193) had a > 50% conversion rate (Fig. 3B). Sites-1 and -2 displayed different results when the C-to-T conversion rates were grouped by the ‘CBE set’. For target site-1, a higher number of T_1_ individuals with high conversion rates (> 50%) were observed in AIDv2 CBE transformants (pCBE-A1 and pCBE-A2) compared to BE3 (pCBE-B1 and pCBE-B2) transformants. Conversely, more T_1_ individuals transformed with BE3 vectors had high conversion rates at position C_6_ of site-2 relative to those transformed with AIDv2 vectors (Fig. 3C). For position C_12_ of site-2, the opposite pattern was observed; the 35S promoter set and the AIDv2 CBE set produced a higher number of T_1_ individuals with high C-to-T conversion rates (Fig. 3B,C).

## Discussion

Genome editing tools can effectively generate stable transgenic lines in plants; however, predicting how efficiently novel mutations can be introduced by particular CRISPR-Cas9 constructs, and how successfully those mutated loci can be transmitted into the next generation, remain as important challenges^23–25^. Recent studies have identified ways to increase the editing efficiency of CRISPR-Cas9 systems in the T_1_ generation, such as using high strength promoters^26–28^ and heat treatment^29^. The 35S promoter, which is often used for overexpression experiments in plant research, can be applied to typical CRISPR/Cas9-mediated experiments in which indel mutations are desired, and germline transmission of the mutant alleles is frequently successful^30^. However, 35S may not be the optimal promoter for base editing systems; the efficiency of base editing is generally lower than that of conventional indel-generating Cas9 systems, and lower rates (ca. 25-fold less) of germline transmission have been reported with 35S promoter use compared to other promoters^15,16,18^.

Our results provide a clear comparative overview of promoter and CBE system use for base editing, which demonstrates that the RPS5a promoter can possibly increase base editing efficiency in cytosine base editing in *Arabidopsis*. However, we cannot rule out the possibility of stochastic bias in this study due to the small number of gRNA target sites used. Nevertheless, our quantitative mutant read assessment via NGS analyses, which included a large number of T_1_ individuals, suggested that RPS5a can be an effective promoter for CBEs. Use of RPS5a showed an increased effect on the CBE efficiency of T_1_ individuals in *Arabidopsis* at at least two cytosine sites and had a higher propensity for generating > 50% mutant reads than the 35S promoter. Given that recessive mutations typically would not manifest when cells carry > 50% normal protein levels, our 50% cutoff for genome editing efficiency was biologically relevant; for example, our data showed that tissues carrying < 50% mutant reads mostly displayed normal-looking phenotypes. Thus, promoters that facilitate high CBE mutation rates in the first generation of plants is desirable, as this would enable the function of genes, or particular residues within genes, to be assessed. Although our results might not provide enough number of tests, we can add the idea that the mutation target site sequence would make various base editing outcomes depending on a CBE system; CBE AIDv2 performed better at site-1, whereas CBE BE3 was more efficient at modifying site-2. This suggests that the two CBEs, which harbor different linkers and deaminase properties, may exhibit different preferences for particular target sequences and/or different fidelities depending on the target sequence composition^9,31,32^ and also the target base position makes differences^4,6^.

Our experimental results prompt us to recommend the use of the RPS5a promoter in vector constructs designed to introduce C-to-T mutations in *Arabidopsis*. We observed that almost 100% C-to-T conversion rates at target site-1 was achievable even in T_1_ generation using pCBE-A2 (pRPS5a-AIDv2; Fig. 1C,D). Additionally, a significant number of T_1_ plants in which site-2 was targeted showed > 50% C-to-T conversion efficiency when transformed with pCBE-B2 (pRPS5a-BE3; Fig. 3); no plants with > 50% conversion rates were observed following transformation with either construct containing the 35S promoter. We would note that CBE choice may not universally guarantee success, as our quantitative assessment showed a bias when multiple cytosines were targeted simultaneously. Furthermore, CBE choice needs to be considered alongside other parameters, such as target site composition, because the active target window for base editing appears to differ between AIDv2 and BE3. Further investigation is required to determine the target preferences of the two CBEs examined in this study, in which a larger set of target sites should be assessed. However, our results do suggest that AIDv2 may function well for a broader, less specific active window of target base changes, whereas BE3 may be more appropriate for the induction of base substitution mutations specifically at positions 4–6 of the active window. It is also evident that both BE3 and AIDv2 are unsuitable for base changes with an active window size of 1 bp, in other words, without concerning bystander editing; for fine precision edits such as this, Prime Editor could be a more appropriate option^33^ or the proper combination of other variants that reduce the active window size^34^ and the extended version of PAM limitation such as x-Cas9^35^, Cas9-NG^36^, near-PAMless SpRY^37^.

Another important factor to consider when designing base editing systems is that unwanted indel inductions by nickase Cas9 activity may arise with the use of CBEs. We observed some indel mutations in our amplicon deep sequencing analysis, but the indel ratios and frequencies in T_1_ individuals were relatively low; 10 of 185 T_1_ individuals harbored indel mutations in site-1 and 11 of 255 T_1_ individuals in site-2, giving an indel mutation rate of > 5%. Notably, 8 of 10 and 6 of 11 cases with indel mutation ratios of > 5% resulted from transformation of pCBE-A2 (pRPS5a-AIDv2) targeting site-1 and pCBE-A1 (p35S-AIDv2) targeting site-2, respectively (Table S2).

In conclusion, our investigation into how promoters and CBE systems can affect the efficiency of cytosine base editing experiments in *Arabidopsis* suggested that the RPS5a promoter can improve CBE vector constructs for *Agrobacterium*-mediated transformation by increasing base editing efficiency. However, the efficiency of cytosine base editing may be further affected by the choice of CBE system, target sequence variability, and the active range of the base pair target window. Nonetheless, the use of the RPS5a promoter is likely to be plausible for cytosine base editing in *Arabidopsis*.

## Methods

### Preparation of vector constructs

All vector constructs used in this study can be found in Fig. 1A. We used the previously published pBAtC^30^ binary vector for the backbone to build pCBE-A1 and pCBE-B1, which contain the 35S promoter and nCas9 (D10A)-PmCDA1-UGI (Addgene plasmid number: 79620) and APOBEC1-XTEN-dCas9 (A840H)-UGI (Addgene plasmid number: 73021), respectively. Similarly, pCBE-A2 and pCBE-B2 were constructed by engaging nCas9 (D10A)-PmCDA1-UGI and APOBEC1-XTEN-dCas9 (A840H)-UGI into the previously published pJY-RpCas9 binary vector^18^, respectively. To clone guide RNAs in each vector we digested vectors with the restriction enzyme, AarI (ThermoFisher Science, USA) and mixed both annealed product of two oligonucleotides and T4 DNA ligase (New England Biolabs, Ipswich, MA, USA) for ligation. These ligation products were transferred to *E. coli* DH5alpha and cells containing proper constructs were selected on the media with spectinomycin. All primers used in this study are listed in Table S1.

### Target selection and gRNA preparation

The two cytosine base editing target sites were manually selected by scanning the NGG (or CCN for antisense strand searching) along the coding region of LFY gene (At5G61850). The CRISPR vector pCBEs were linearized by the restriction enzyme AarI for 3 h to produce 4 bp overhangs, then purified using the ExpinTM PCR SV mini kit (GeneAll®, Cat# 103–202). Forward and reverse oligos (site-1 and site-2) for each guide sequence were mixed with T4 DNA ligase 10X buffer (New England BioLabs®, Cat# B0202S) to 10 μM each and annealed using the following thermocycling program: pre-denaturation for 1 min at 95 °C; 95–25 °C for − 1 °C/min; and hold at 10 °C. The linearized vectors and annealed oligos for guide sequences were ligated and transformed into *E*. *coli* competent cells as previously described^30^. *E*. *coli* transformants were selected on spectinomycin; plasmid DNA was isolated using the ExprepTM plasmid SV mini kit (GeneAll®, Cat# 101–102) and validated by Sanger sequencing.

### Agrobacterium-mediated transformation

The *Arabidopsis thaliana* Col-0 ecotype was used for *Agrobacterium tumefaciens* GV3101-mediated plant transformation via floral dip^28^. Col-0 plants were grown under long day conditions (16 h light/8 h dark) at 22 °C in a growth room (Koencon, Hanam, South Korea). Light was generated from a 32 W Osram lamp (170 mmol/2 m/s).

### Targeted deep sequencing and mutation pattern analysis

Genomic DNA for targeted deep sequencing analysis was extracted from two or more randomly selected leaves from each individual of T_1_ or T_2_ plants using commercially available plant DNA extraction kit. The on-target sequence was amplified from genomic DNA using target-specific primers (Table S1) that were designed using reference genome sequences and attached with adapter sequences. Multiplexing indices and sequencing adaptors were added by an additional PCR using the protocol supplied from the sequencing company, Macrogen (Seoul, South Korea). High-throughput sequencing was performed using Illumina Mini-seq (San Diego, CA, USA) with the paired-end multiplexed library. Raw reads from paired-end Mini-seq sequencing were joined by the program, ‘fastq-join’ and implemented in the package, ‘eu-util’ (http://code.google.com/p/ea-utils). Base substitution rates were calculated using the ‘BE-analyzer’ on the web-based program (http://rgenome.net) or an in-house python script. The average amplicon read number generated in this study was 11,137 and can be found in Table S2. We also extracted the DNA from several randomly selected leaves of T_2_ individuals and amplified the on-target region using the same primer sets used in the targeted deep sequencing. The PCR product was directly sequenced by Macrogen.

### Detection of off-target mutations

Three transgenic T2 plants by pCBE-A1 and two by pCBE-B2 on target site-2 were selected for each off-target detection. Potential off-target sites allowing 3 bp mismatches on target sequence were searched using Cas-Offinder^22^ based on the Arabidopsis reference genome and amplified with the primer sets listed in supplementary Fig. S2. Purified PCR products were sent for Sanger sequencing (Macrogen, Seoul, South Korea) to identify base substitution.

## Supplementary Information

Supplementary Information 1.

Supplementary Information 2.
