# Supplementary material for: The efficacy of CRISPR-mediated cytosine base editing with the RPS5a promoter in Arabidopsis thaliana
Source: Sci Rep. 2021 Apr 13;11:8087. doi: 10.1038/s41598-021-87669-y (PMC8044221; doi:10.1038/s41598-021-87669-y)

The efficacy of CRISPR-mediated cytosine base editing with the RPS5a promoter in *Arabidopsis thaliana*

Minkyung Choi[1,†], Jae-Young Yun[1,2,†], Jun-Hyuk Kim[3], Jin-Soo Kim[1,*], and Sang-Tae Kim[1,3,*]

Supplementary Information

Table S1: Primer sequence list for amplicon deep sequencing, vector cloning, and off-target detection.

Table S2: Targeted deep sequencing analysis for all T_1 plants (in the Excel file).

Figure S1: PCR validation for T-DNA left-over in T_2 plants. Priming sites and PCR results with primer sequences used in the experiment were presented.

Figure S2: Potential off-target sites detection. Primer sets and Sanger sequencing were presented.

Supplementary information

Table S1 Primer sequence list for amplicon deep sequencing, vector cloning, and off-target detection.

Primer list for amplicon deep sequencing.

name	sequences (5'-3')
site-1,2 _F	ATGGATCCTGAAGGTTTCACG
site-1,2 _R	CCTTTCCCTAACTCGACACG
site-1_target_F	ACACTCTTTCCCTACACGACGCTCTTCCGATCTGTGGGTATGAAGGACGAGGA
site-1_target_R	GTGACTGGAGTTCAGACGTGTGCTCTTCCGATCTGGCGGAGAGTAGCAAATGAC
site-2_target_F	ACACTCTTTCCCTACACGACGCTCTTCCGATCTTGGACAGGGTTATCTGAGGAA
site-2_target_R	GTGACTGGAGTTCAGACGTGTGCTCTTCCGATCTGTCGTCATCCTCACCTTCGT

Primer list for vector cloning

name	sequences (5'-3')
pCBE-A1_F	ACAAAAAAGCAGGCTATGGCACCGAAGAAGAAGC
pCBE-A1_R	CAAGAAAGCTGGGTGTCAGAAGAACTCGTCAAGAAGG
pCBE-B1_F	AAAGCAGGCTTCATGAGCTCAGAGACTGGCCCAG
pCBE-B1_R	CAAGAAAGCTGGGTGTTAGGCGTAGTCGGGCAC
pCBE-A2_F	TCACCACAGCCCTAGGATGGCACCGAAGAAGAAGC
pCBE-A2_R	TTCATATCCCCTCGAGTCATAGCATCTTGATCTTGTTCTCT
pCBE-B2_F	TCACCACAGCCCTAGGATGAGCTCAGAGACTGGCCCA
pCBE-B2_R	TCATATCCCCTCGAGTTAGACTTTCCTCTTCTTCTTGGG

Primer list for off-target detection

target	primer name	5'-3' seq
Site-2_OT01	Deep0603	TACTGTGTGCCACAAGGTTT
Site-2_OT01	Deep0604	AATGGGTCTGTTGCTTTGGA
Site-2_OT02	Deep0605	ACTTCCATCAACACGACCTC
Site-2_OT02	Deep0606	ACCCGAGGTAAGAGACTACC
Site-2_OT03	Deep0607	GATCAAATCGGATCGGGTGA
Site-2_OT03	Deep0608	ACACCAAGAGAGGCAAAACA
Site-2_OT04	Deep0609	TGGCTCTTTATGGGTTGCAT
Site-2_OT04	Deep0610	AAATTGCCTGGTATGCCGAT
Site-2_OT05	Deep0611	CGTACCATTCTGACAAATCTCG
Site-2_OT05	Deep0612	AACGAGCTCCTGCACTTTTA
Site-2_OT06	Deep0613	ACAGTGCACGAAAAATCGAG
Site-2_OT06	Deep0614	AAACCACACCACACTCCAAA
Site-2_OT07	Deep0615	GAAGTCCATGCGGAATCTCA
Site-2_OT07	Deep0616	GCTGTCCAAGGATCTCAACA
Site-2_OT08	Deep0617	AGAATGTGATCTTCCAGCGG

target	primer name	5'-3' seq
Site-2_OT08	Deep0618	CACCAACAACAACAACTGCA
Site-2_OT09	Deep0619	TGGGAGCCTGGAAAAATTGT
Site-2_OT09	Deep0620	AAGACAAAAAGCCAGACGGA
Site-2_OT10	Deep0621	ACATGGTTCGGTTTGCAATG
Site-2_OT10	Deep0622	GAAAACACCACTTTGGAGGC
Site-2_OT11	Deep0623	GCCTTTCATTGGAGTGGTCT
Site-2_OT11	Deep0624	TGCTGCATGTGACATGTACT
Site-2_OT12	Deep0625	CATGATCCTTGGTCTGTCCC
Site-2_OT12	Deep0626	TTGCTTGAGGTTGAACCCAT
Site-2_OT13	Deep0627	AGCCGAGTCTAACAGAGTCA
Site-2_OT13	Deep0628	ATTGACGGGAACAGAAGTCC
Site-2_OT14	Deep0629	GTGGTTGAGGAGATGTGTGT
Site-2_OT14	Deep0630	TGGGACATGTGCTTTTTGAAC
Site-2_OT15	Deep0631	AGCAGGACAAGGTTCAGTTC
Site-2_OT15	Deep0632	AGACCAAAACACACAGAGCA
Site-2_OT16	Deep0633	GGCCTCCGAATTCTTCATCA
Site-2_OT16	Deep0634	GGGATGAGTGTCTCTCTTGC
Site-2_OT17	Deep0635	TCGTTCGTGTAATCACTGCA
Site-2_OT17	Deep0636	GTCGCTTGTTCGAATTGCAT
Site-2_OT18	Deep0637	CAGGGTAATGGCCTCTCATG
Site-2_OT18	Deep0638	GCAGAGTCACAGCAGATGAT

Supplement information

Figure S1. PCR validation for T-DNA left-over in T_2 plants

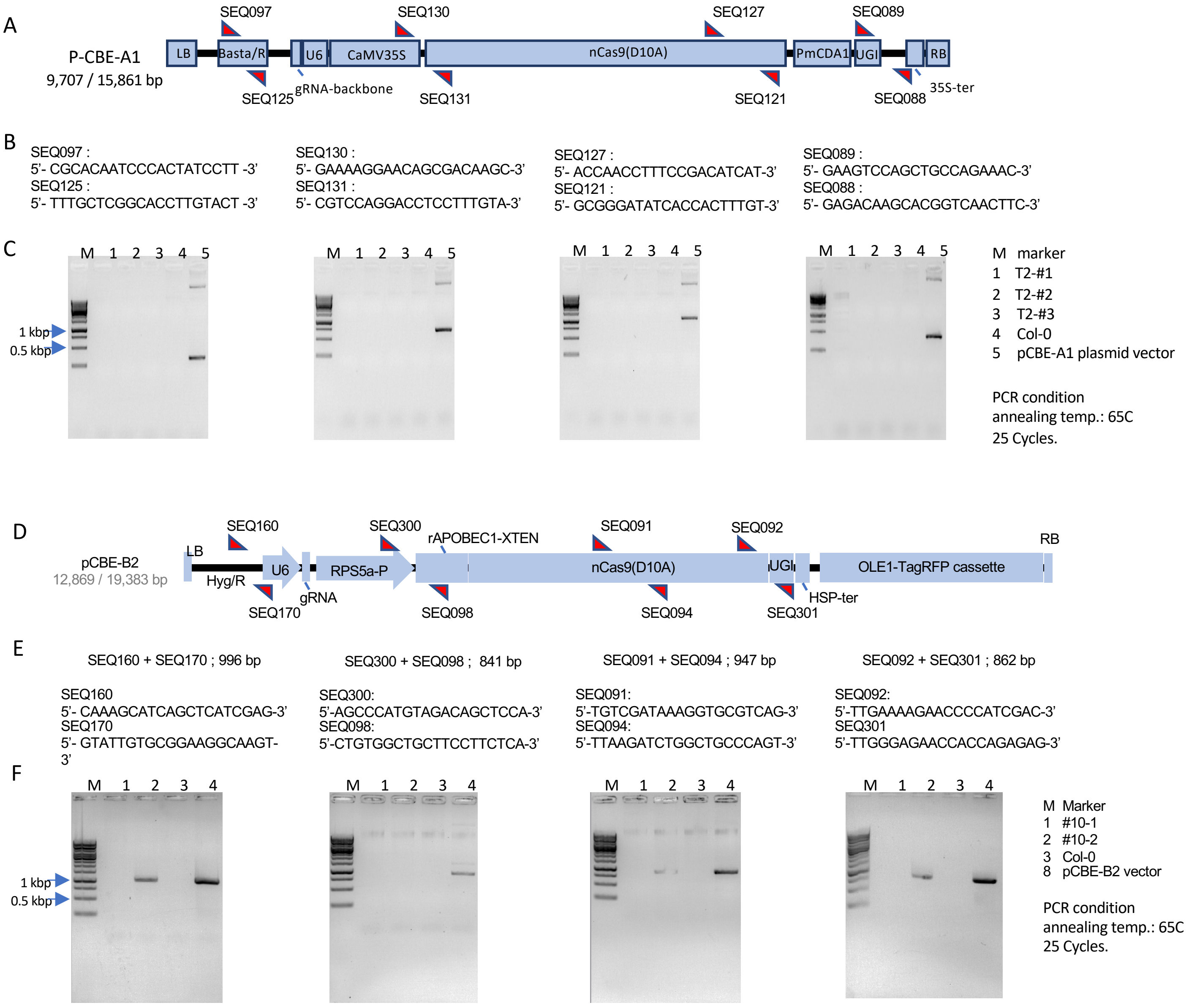

Figure S1. A. The simplified vector construct of T-DNA plasmid (pCBE-A1) showing T-DNA remnant confirmation PCR primer sets (red triangles indicated with names). B. Primer sets used for amplification. C. Electrophoresis of PCR products for selected T_2 plants (3 individuals) showing leafy phenotype, WT:Col-0, and pCBE-A1 vector plasmid. The summary of PCR conditions is presented. D. The simplified vector construct of T-DNA plasmid (pCBE-B2) showing T-DNA remnant confirmation PCR primer sets (red triangles indicated with names). E. Primer sets used for amplification. F. Electrophoresis of PCR products for selected T_2 plants (2 individuals) showing leafy phenotype, WT:Col-0, and pCBE-B2 vector. The summary of PCR conditions is presented.

Supplement information:

Figure S2. Potential off-target to target site-2 (5'-AGAAG$\mathbf{C}_{6}$AA$\mathbf{C}_{9}$AG$\mathbf{C}_{12}$AG$\mathbf{C}_{15}$AGAGACGG-3') validations by Sanger-sequencing for T_2 plants; A) #1, #2, #3 transformed by pCBE-A1 and B) #10-1, #10-2 by pCBE-B2 in a top-down order respectively.

Potential off-target 001
Reference position: chr4_16604925, Mismatch number: 2
Target sequence: AGAAGtAACAGCAGgAGAGACGG (mismatched nucleotide: low case)

A) reference seq., #1, #2, #3

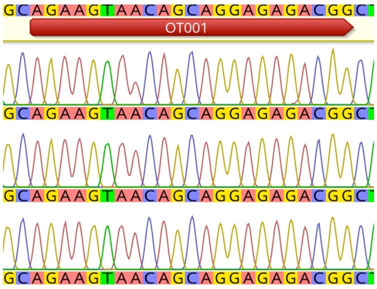

B) reference seq., #10-1, #10-2

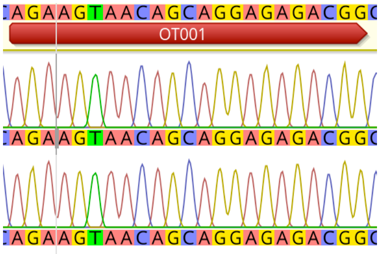

Potential off-target 002
Reference position: chr5_18711842, Mismatch number: 2
Target sequence: tGAAGCAgCAGCAGCAGAGAAGG (mismatched nucleotide: low case)

A) reference seq., #1, #2, #3

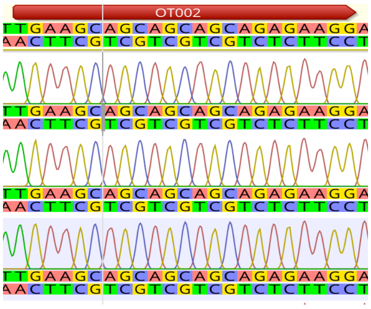

B) reference seq., #10-1, #10-2

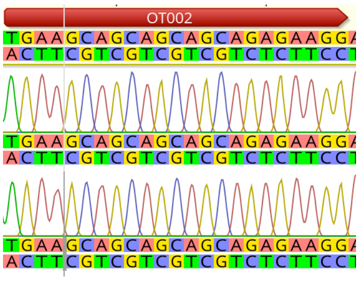

Potential off-target 003
Reference position: chr1_21142015, Mismatch number: 3
Target sequence: AGAAGgAtCAGaAGCAGAGATAG (mismatched nucleotide: low case)

A) reference seq., #1, #2, #3

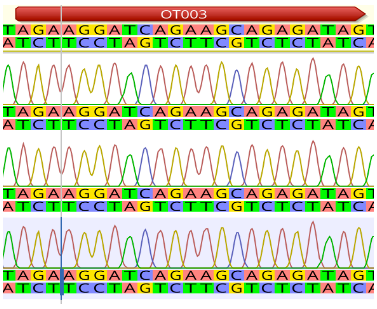

B) reference seq., #10-1, #10-2

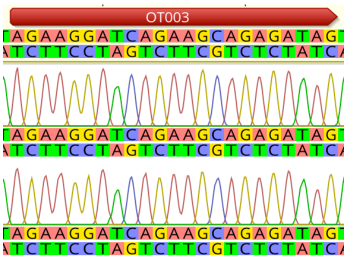

Potential off-target 004

Reference position: chr1_2594759, Mismatch number: 3

Target sequence: AGttGCAACAGCAtCAGAGACAG (mismatched nucleotide: low case)

A) reference seq., #1, #2, #3

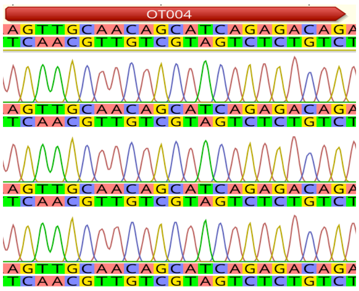

B) reference seq., #10-1, #10-2

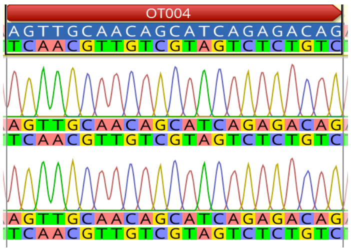

Potential off-target 005

Reference position: chr1_27979219, Mismatch number: 3

Target sequence: AGAAGaAACAGCAGCAGAtgAAG (mismatched nucleotide: low case)

A) reference seq., #1, #2, #3

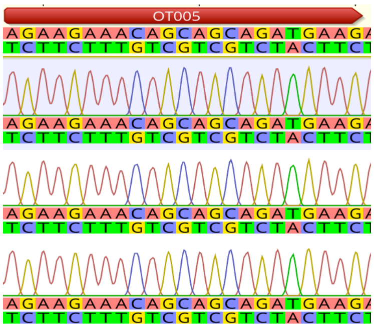

B) reference seq., #10-1, #10-2

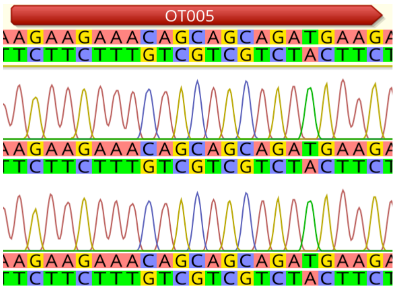

Potential off-target 006

Reference position: chr1_3323131, Mismatch number: 3

Target sequence: AGAAGCAACAGaAGCAagGAGGG (mismatched nucleotide: low case)

A) reference seq., #1, #2, #3

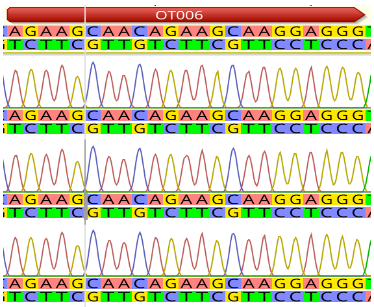

B) reference seq., #10-1, #10-2

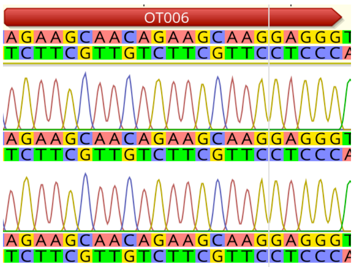

Potential off-target 007

Reference position: chr1_3613138, Mismatch number: 3

Target sequence: AGgAGCAACAaCtGCAGAGACAG (mismatched nucleotide: low case)

A) reference seq., #1, #2, #3

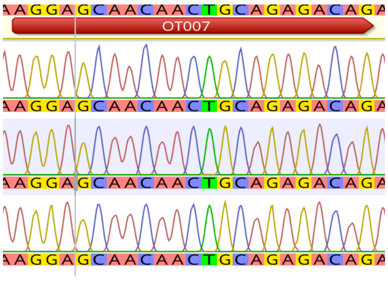

B) reference seq., #10-1, #10-2

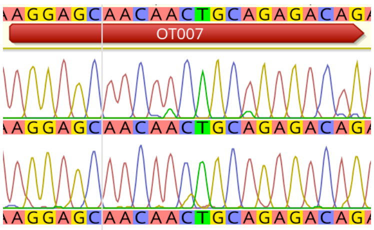

Potential off-target 008

Reference position: chr1_6629853, Mismatch number: 3

Target sequence: AGAAGCtACAGCAGCAGcaACAG (mismatched nucleotide: low case)

A) reference seq., #1, #2, #3

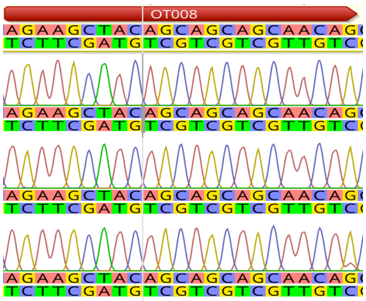

B) reference seq., #10-1, #10-2

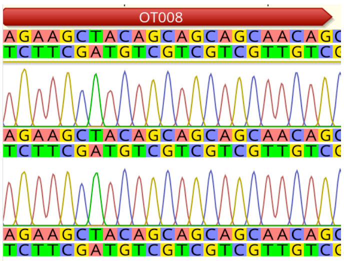

Potential off-target 009

Reference position: chr2_10911998, Mismatch number: 3

Target sequence: AcAAGCAAaAGCAGaAGAGATGG (mismatched nucleotide: low case)

A) reference seq., #1, #2, #3

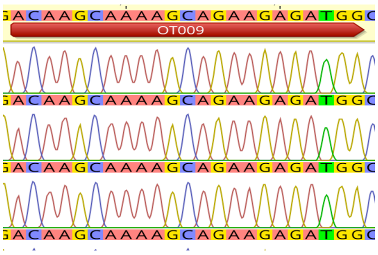

B) reference seq., #10-1, #10-2

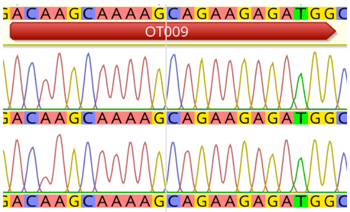

Potential off-target 010

Reference position: chr2_11584413, Mismatch number: 3

Target sequence: AGttGCAACAGCAtCAGAGACAG (mismatched nucleotide: low case)

A) reference seq., #1, #2, #3

B) reference seq., #10-1, #10-2

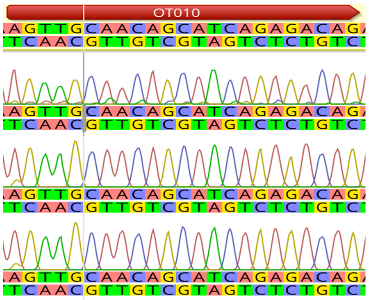

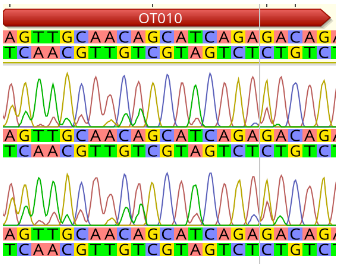

Potential off-target 011

Reference position: chr2_14103022, Mismatch number: 3

Target sequence: AGAAGCAgCAGCAGCAGAttTGG (mismatched nucleotide: low case)

A) reference seq., #1, #2, #3

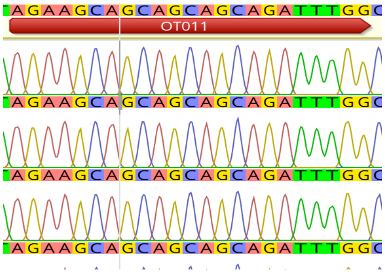

B) reference seq., #10-1, #10-2

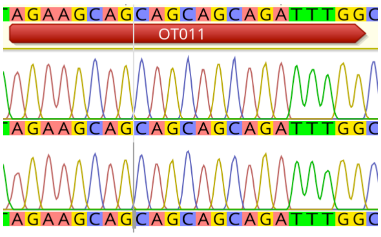

Potential off-target 012

Reference position: chr2_508450, Mismatch number: 3

Target sequence: AGAAGaAACAGCAGCAGcGgCGG (mismatched nucleotide: low case)

A) reference seq., #1, #2, #3

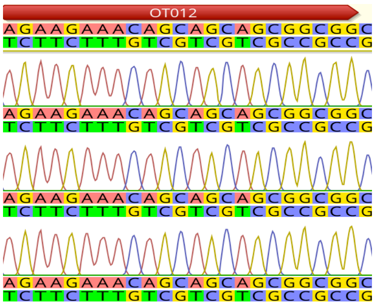

B) reference seq., #10-1, #10-2

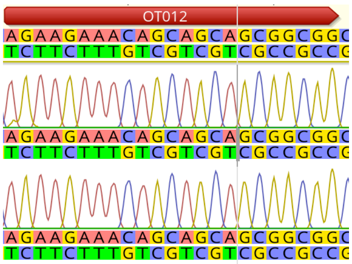

Potential off-target 013

Reference position: chr3_22175095, Mismatch number: 3

Target sequence: gGAAGCAAgAGaAGCAGAGAGAG (mismatched nucleotide: low case)

A) reference seq., #1, #2, #3

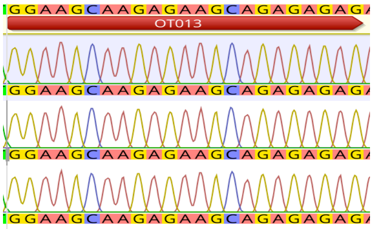

B) reference seq., #10-1, #10-2

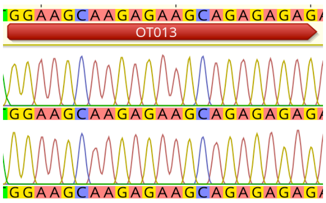

Potential off-target 014

Reference position: chr3_8647531, Mismatch number: 3

Target sequence: AGAAGaAACAGaAGCAaAGAGAG (mismatched nucleotide: low case)

A) reference seq., #1, #2, #3

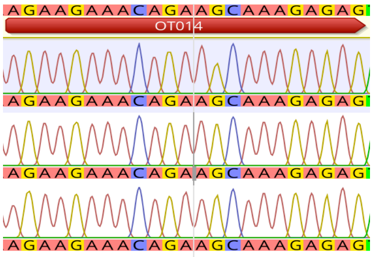

B) reference seq., #10-1, #10-2

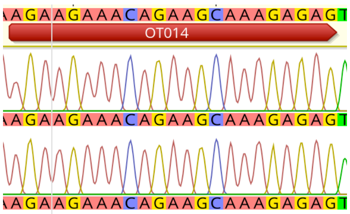

Potential off-target 015

Reference position: chr4_13028612, Mismatch number: 3

Target sequence: AGAAaCAgCAGCAGCtGAGACAG (mismatched nucleotide: low case)

A) reference seq., #1, #2, #3

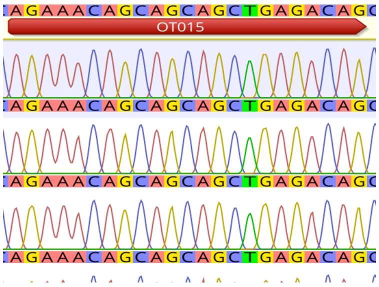

B) reference seq., #10-1, #10-2

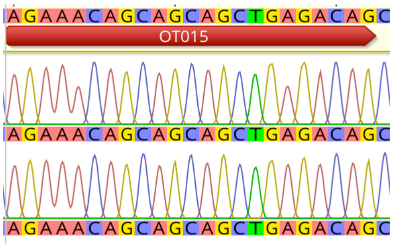

Potential off-target 016

Reference position: chr5_20957198, Mismatch number: 3

Target sequence: AGcAGCAgCAGCAGCAGAtAAAG (mismatched nucleotide: low case)

A) reference seq., #1, #2, #3

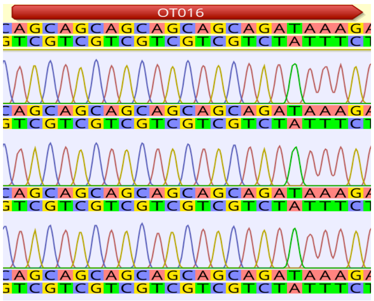

B) reference seq., #10-1, #10-2

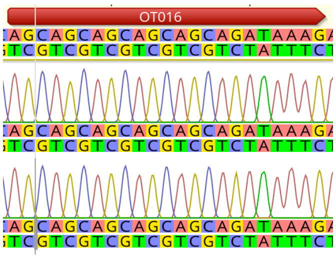

Potential off-target 017

Reference position: chr5_25728814, Mismatch number: 3

Target sequence: gGAAGCAACAGCAGCgGAGgAAG (mismatched nucleotide: low case)

A) reference seq., #1, #2, #3

B) reference seq., #10-1, #10-2

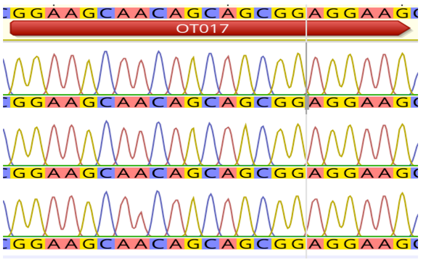

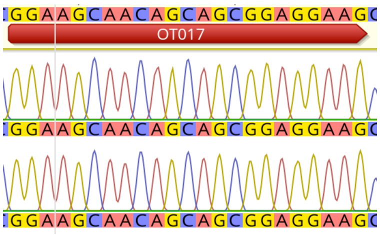

Potential off-target 018

Reference position: chr5_8886844, Mismatch number: 3

Target sequence: _AcAAGCAAaAGCAGaAGAGATGG (mismatched nucleotide: low case)

A) reference seq., #1, #2, #3

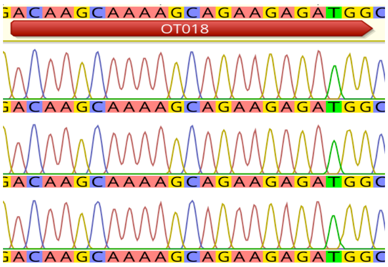

B) reference seq., #10-1, #10-2

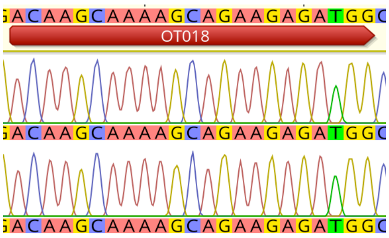

Supplement: Supplementary file 1 — Supplementary Information 1. [file 41598_2021_87669_MOESM1_ESM.pdf]
